# Role of RDW in Prediction of Burn after Caustic Substance Ingestion

**DOI:** 10.3390/children5010005

**Published:** 2017-12-29

**Authors:** Emrah Aydin, Omer Faruk Beser, Soner Sazak, Ensar Duras

**Affiliations:** 1 Department of Pediatric Surgery, Bahcelievler State Hospital, 34186 Istanbul, Turkey; 2 Cincinnati Children’s Hospital Medical Center, 3333 Burnet Avenue, Cincinnati, OH 45229-3039, USA; 3 Department of Pediatric Gastroenterology, Okmeydani Education & Training Hospital, 34384 Istanbul, Turkey; omer.beser@saglik.gov.tr; 4 Department of Pediatrics, Okmeydani Education & Training Hospital, 34384 Istanbul, Turkey; soner.sazak@saglik.gov.tr (S.S.); ensar.duras@saglik.gov.tr (E.D.)

**Keywords:** caustic substance ingestion, esophageal burn, RDW

## Abstract

A quantifiable, quick, inexpensive and reproducible predictor is needed to decide if caustic substance ingestion results in burn regardless of the symptoms. A multicenter cohort study was conducted to investigate the predictive value of red cell distribution width (RDW) in detecting the esophageal burns. The data of 174 patients were retrospectively analyzed. Eleven patients were excluded due to inability to define the substance ingested. Complete blood count (CBC) was taken at admission, and an esophagogastroduodenoscopy was performed within the first 12–24 h in all patients, regardless of their symptoms. The age and gender of the patients, the types of substances ingested, the parameters in the CBC and the severity of the esophageal injury were correlated. Esophageal burns were diagnosed in 38 of 163 patients (23.3%). The risk of esophageal burn with RDW values below 12.20 was significantly lower. Multivariate analysis showed that RDW was the most significant predictor of esophageal burn (*p* = 0.000, odds ratio (OR) 7.74 (95% confidence interval (CI), 3.02–19.9)). Receiver operating characteristic (ROC) curve analysis demonstrated 84.2% sensitivity at a cut-off value of 12.20 for RDW. The results showed that CBC parameters could avoid unnecessary esophagogastroduodenoscopy. The RDW values regardless of the symptomatology is a good predictor of esophageal burns, and an RDW value over 12.20 shows the increased risk of esophageal burn.

## 1. Introduction

Although caustic substance ingestion (CSI) used to be a big problem in developed countries, it is still a life-threatening problem worldwide, mostly in developing countries. In the USA, approximately 5000–15,000 cases occur yearly [1]. Female to male ratio is 2/3 in CSI [2] Esophagogastroduodenoscopy (EGD) is the gold standard for the diagnosis. Although many patients are faced every day in the emergency departments, studies published in the literature conflict with each other in the diagnosis [1,3,4]. Per the hazard caused by CSI is enormous, the ratio of unnecessary EGDs is between 60% and 82% in the literature [2,4]. This high number of false negative results is due to discordance of the signs and symptoms of the presence of burn. Therefore, a quantifiable, quick, inexpensive and reproducible predictor is needed to decide if the CSI resulted in burn or not.

There are studies in the literature demonstrating the relation between inflammation and markers in complete blood count (CBC); such as red blood cell distribution width (RDW), mean corpuscular volume (MPV), platelet distribution width (PDW), lymphocyte and neutrophil counts. Red cell distribution width, a quantitative measurement of variability and size of the erythrocytes, has emerged as either a diagnostic or a prognostic factor in many diseases such as coronary artery disease, epithelian ovarian cancer and acute pancreatitis [5,6,7,8,9,10,11,12]. Herein, we presented a study that demonstrates RDW as a novel diagnostic marker in the diagnosis of burns in CSI before EGD.

## 2. Materials and Methods

This multicenter retrospective cohort analysis consecutively enrolled a series of patients with CSI who were admitted to the emergency, pediatric gastroenterology, and pediatric surgery departments and consented for the treatment between January 2015 and April 2016. Bahcelievler State Hospital Institutional Review Board approval was obtained (ethical approval number is #2016-2615), and data was assembled through an institutional database and augmented with the electronic medical record for the hospital. There were 174 patients, 11 of whom were excluded due to ingested substances those could not be identified. The identification of the substances was made by contacting with national poisoning center. A CBC was taken at the admission, and an EGD was performed in all patients within 12–24 h after ingestion of caustic substance regardless of their symptomatology. Esophageal burns were classified by using an endoscopic classification modified by Zargar [13].

Statistical analysis was performed with IBM SPSS Statistics 20.0.0 (Chicago, IL, USA). The characteristics of the study sample were summarized by descriptive statistics. Kolmogorov-Smirnov test was used to demonstrate normal distribution. One-Way analysis of variance (ANOVA) was used for homogeneity of the variables, Student’s *t*-test and Pearson correlation were used for parametric data. Multivariate logistic regression analyses were used to assess whether the markers are independent to predict burn. Receiver operating characteristic (ROC) curves were used to assess the accuracy of the markers. Statistical associations were considered significant if the *p*-value was <0.05.

## 3. Results

Out of 174 potential subjects, 163 patients (96.7%) enrolled in the study because the substances ingested could not be identified in 11 patients. Seventy-four patients (45.4%) were female. A substantial majority of the patients (88.3%) were below 6 years of age with a median age of 2.00 ± 3.42 years at the time of admission. All cases of ingestion were accidental, and 75.5% of the patients admitted to the hospital within the first 12 h of ingestion. The overwhelming majority of the substances ingested were alkali (69.9%). The esophageal burn was diagnosed in 38 cases (23.3%), 2 of which were graded as severe (third degree). No other accompanying lesion of the gastrointestinal system was documented during the EGD, and no complications were encountered during the procedure. The severity of the esophageal burn lesion was increased at older ages which, however, was not statistically significant (*p* = 0.523) (Table 1). Age, gender and type of substances ingested were comparable between groups (Table 2).

**Figure 1:**
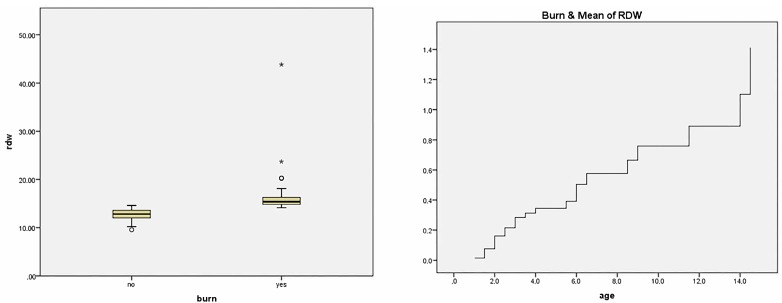
Distribution of red cell distribution width (RDW) between groups and proportional hazards (COX) regression hazard analysis.

The comparison of the parameters of CBC was summarized in Table 3. The mean white blood cell (WBC), platelet (plt), lymphocyte (lym), neutrophil (neut) and eosinophil counts (eos) were decreased in the patients with esophageal burns, while RDW, MPV, PDW, monocyte (mono), basophil (baso) counts and neutrophil-lymphocyte ratio were increased. Among all, the only parameter that was significantly different was RDW (odds ratio (OR) 7.74 (95% confidence interval (CI), 3.02–19.9)). The correlation coefficient for RDW and existence of burn was 0.718 (*p* = 0.000). Univariate COX regression hazard (proportional hazards) analyses showed RDW values significantly higher in patients with an esophageal burn after CSI (Figure 1). ROC curve analysis was done to optimize cut-off for RDW, and the cut-off was found as 12.20. The sensitivity of the RDW was 84.2%, specificity was 59.2%, the positive predictive value was 38.6%, and the negative predictive value was 92.5%.

All children without a burn were discharged at postoperative 4–6 h. Among the patients with esophageal burns, 24 patients (63.2%) had repeat EGD at post-procedure day 7 regardless of their symptoms. Two of these patients were demonstrated to have esophageal strictures on the upper gastrointestinal system contrast series performed two weeks after discharge and required esophageal balloon dilatation.

## 4. Discussion

Studies in the literature aiming to demonstrate the predictability of esophageal injury depend mostly on presence or absence of symptoms and yet they are conflicting [3,4]. When the degree of burn is severe, which constitutes a small number of the patient group, the predictability of burn is statistically significant. However, there was no statistical significance between patients without burn and patients with mild and moderate burns regarding the presence or absence of symptoms [4]. In a study done by Betalli et al. reported two patients out of 19 with third-degree esophageal burn but without symptoms, even they concluded to avoid EGD in asymptomatic patients. They also stated that the risk of severe damage increases in proportion to the number of signs and symptoms [4].

Many studies in the literature related with CSI including this one have 60–80% false EGD rates, and yet many efforts have been put onto predicting esophageal burn at admission [1,2]. One major problem in the determination of the esophageal burn after CSI is the reliability of the history gathered from parents. This is either because there is no witness by an adult, or the parents are too anxious to recognize the signs and symptoms which let the EGD inevitable. Therefore, an objective parameter that would predict the presence of burn before EGD is needed to prevent this discrepancy.

Red cell distribution width, a quantitative measurement of variability and size of the erythrocytes, is routinely reported in CBC, widely available, inexpensive and highly reproducible test. It’s calculated by dividing the MPV by the standard deviation of erythrocytes and then multiplied for 100, to show the data as a percentage. Although it has long been used for differential diagnosis of anemias, it has emerged as a new risk marker for many different diseases associated with acute and chronic inflammation [5,6,7,8,9,10,11,12].

Our study has some fundamental results. First, RDW was significantly higher in patients with an esophageal burn after CSI compared to the ones that did not. Second, RDW was found to be an independent predictor of esophageal burn in multivariate logistic regression analyses. We also demonstrated that RDW values after CSI, with cut-off 12.20 determined by ROC curve, acts as a predictor for esophageal burns. We speculate that inflammation caused by caustic substance may shorten the half-life of the erythrocytes, change the membrane characteristics and cause an increase in RDW values.

The age and the gender distribution between groups were equal. More than 2/3 of the patients in both groups were ingested alkali type of caustic substance. The mean WBC count and platelet count were higher in patients without burn. However, it was not statistically significant. Although the mean values of RDW, MPV, PDW and neutrophil/lymphocyte ratio were higher in patients with esophageal burn, there was statistical significance only with RDW values. It is unlikely that patients with esophageal burns had other causes for elevated RDW such as iron deficiency or folate deficiency because no patients had anemia and all had normal mean corpuscular volume (MCV) values.

Our study has some weaknesses. First, it was a retrospective cohort analysis. Second, we had a small number of patient population with burns, specifically severe esophageal burns which might be the reason why other parameters such as MPV and neutrophil/lymphocyte ratio was not statistically significant although they were higher. On the other hand, this study is the first one in the literature to show the correlation between RDW values and esophageal burns after CSI.

Even though EGD can distinguishes patients with burns after CSI, it requires patients to be sedated in the operating room and carries the risk of iatrogenic injury. Therefore, many physicians in the developed world believe it is unnecessary to perform endoscopic examination in asymptomatic patients after accidental CSI. It has been the physician’s responsibility to find a balance between these. This study has promising results that suggest RDW might help identify those patients with caustic ingestion who have an increased risk of esophageal burns, and yet it is an inexpensive, convenient and widely available marker. Because of the retrospective nature of this study along with the small numbers of patients involved and limited number of severe burns, a prospective study looking at a larger patient size would be needed.

## Figures and Tables

**Table 1:** The mean age of the patients per burn degree.

		Age	95% CI
No burn		3.29 ± 3.21	2.72–3.86
Burn	Grade 1	3.73 ± 3.59	1.31–6.14
	Grade 2	4.32 ± 4.55	2.30–6.34
	Grade 3	5.25 ± 4.60	−36.05–46.55

Values expressed as means ± standard deviations, CI: confidence interval.

**Table 2:** Demographic features of the patients.

	Burn (+)	Burn (−)	*p*
age	4.03 ± 4.04	3.29 ± 3.21	0.246
gender			0.925
male	21 (55.3%)	68 (54.4%)	
female	17 (44.7%)	57 (45.6%)	
type of substance			0.742
acid	12 (31.6%)	36 (28.8%)	
base	26 (68.4%)	89 (71.2%)	

Values expressed as means ± standard deviations or count (percentage of group).

**Table 3:** Laboratory values of the patients.

	Burn (+)	Burn (−)	*p*
wbc	10,760.26 ± 3211.55	11,192.11 ± 3602.84	0.508
plt	337,157.89 ± 78,561.46	341,478.16 ± 91,441.24	0.793
hgb	12.98 ± 1.13	12.84 ± 1.40	0.575
htc	35.90 ± 3.07	37.87 ± 3.66	0.704
mcv	85.28 ± 4.26	84.23 ± 4.17	0.153
rdw	14.63 ± 5.45	11.99 ± 1.40	0.000 *
mpw	8.61 ± 1.69	8.39 ± 1.60	0.478
pdw	13.86 ± 4.47	13.44 ± 4.23	0.606
lym	5.35 ± 2.42	5.44 ± 2.31	0.835
mono	0.86 ± 0.31	0.83 ± 0.30	0.600
neut	4.19 ± 1.7	4.55 ± 2.71	0.451
eos	0.32 ± 0.23	0.33 ± 0.36	0.850
baso	0.11 ± 0.2	0.11 ± 0.17	0.882
neut/lym	1.05 ± 0.86	1.04 ± 0.98	0.930

Values expressed as means ± standard deviations, * (95% CI 12.83–16.42), wbc: white blood cell, plt: platelet, hgb: hemoglobin, mcv: mean corpuscular volume, rdw: red cell distribution width, mpw: mean platelet volume, pdw: platelet distribution width, lym: lymphocyte, mono: monocyte, neut: neutrophil, eos: eosinophil, baso: basophil.
